# Inhibitory Effects of Palm Tocotrienol-Rich Fraction Supplementation on Bilirubin-Metabolizing Enzymes in Hyperbilirubinemic Adult Rats

**DOI:** 10.1371/journal.pone.0089248

**Published:** 2014-02-20

**Authors:** Yusof Kamisah, Jing Jye Lim, Chew-Lian Lim, Ahmad Y. Asmadi

**Affiliations:** 1 Department of Pharmacology, Faculty of Medicine, UKM Medical Center, Universiti Kebangsaan Malaysia, Kuala Lumpur, Malaysia; 2 Department of Biomedical Sciences, Faculty of Health Sciences, Universiti Kebangsaan Malaysia, Kuala Lumpur, Malaysia; 3 Faculty of Traditional and Complementary Medicine, Cyberjaya University College of Medical Sciences, Cyberjaya, Selangor, Malaysia; University of Catania, Italy

## Abstract

**Background:**

Phenylhydrazine, a hemolytic agent, is widely used as a model of experimental hyperbilirubinemia. Palm tocotrienol-rich fraction (TRF) was shown to exert beneficial effects in hyperbilirubinemic rat neonates.

**Aim:**

To investigate the effects of palm TRF supplementation on hepatic bilirubin-metabolizing enzymes and ocidative stress status in rats administered phenylhydrazine.

**Methods:**

Twenty-four male Wistar rats were divided into two groups; one group was intraperitoneally injected with palm TRF at the dose of 30 mg/kg/day, while another group was only given vehicle (control) (vitamin E-free palm oil) for 14 days. Twenty-four hours after the last dose, each group was further subdivided into another two groups. One group was administered phenylhydrazine (100 mg/kg, intraperitoneally) and another group was administered normal saline. Twenty-four hours later, blood and liver were collected for biochemical parameter measurements.

**Results:**

Phenylhydrazine increased plasma total bilirubin level and oxidative stress in the erythrocytes as well as in the liver, which were reduced by the pretreatment of palm TRF. Palm TRF also prevented the increases in hepatic heme oxygenase, biliverdin reductase and UDP-glucuronyltransferase activities induced by phenylhydrazine.

**Conclusion:**

Palm tocotrienol-rich fraction was able to afford protection against phenylhydrazine-induced hyperbilirubinemia, possibly by reducing oxidative stress and inhibiting bilirubin-metabolizing enzymes in the liver.

## Introduction

Phenylhydrazine is a strong oxidant agent, which is extensively used in industries, laboratories and therapeutics [1]. It may induce toxic effects towards human health, such as hemolytic anemia, inflammation and alteration in the liver [2], [3]. The autooxidation of phenylhydrazine generates reactive oxygen species (ROS) and some phenylhydrazine-derived radicals [4], which further promote an increase in oxidative stress [5].

Phenylhydrazine-induced hemolysis increases the formation of bilirubin [6] and would affect the enzymes that are involved in the bilirubin metabolism. Heme, a degradation product of hemolysis will be oxidized and converted into biliverdin by heme oxygenase, a rate-limiting enzyme for bilirubin synthesis. Biliverdin is then reduced to bilirubin by biliverdin reductase before being conjugated to glucuronides by UDP-glucuronyltransferase in the liver [7]. Another enzyme involved in the bilirubin metabolism is aminolevulinic acid synthase which is affected by the increased heme production [8]. Phenylhydrazine was shown to increase aminolevulinic acid synthase [8] and heme oxygenase activities in the rat liver [9].

The increase in plasma bilirubin level can be reduced by antioxidants [10], [11]. Palm tocotrienol-rich fraction (TRF), an antioxidant extract from palm oil, was also reported to be protective against experimental hyperbilirubinemia [12], [13]. It contains both tocopherol and tocotrienol at the ratio of 1∶3.Tocopherol is more widespread in plants compared to tocotrienol which the latter being more abundant in palm oil. Tocotrienol differs from tocopherol by the presence of an unsaturated side chain [14].

Due to the prooxidant activity of phenylhydrazine, we postulated that palm tocotrienol-rich fraction might counteract the detrimental effect of the chemical. Therefore, the aim of the present study was to investigate the effects of palm TRF supplementation on oxidative stress and hepatic bilirubin metabolism in rats given phenylhydrazine.

## Materials and Methods

### Chemicals and Experimental Animals

All chemicals and enzymes were purchased from Sigma-Aldrich Chemical Co. (St. Louis, MO, USA), unless indicated otherwise. The palm tocotrienol-rich fraction was a generous gift from Dr Ab Gapor Mat Top (Malaysian Palm Oil Board) and it was prepared according to the method of Gapor et al. [15]. Its composition was 21% α-tocopherol, 17% α-tocotrienol, 4% γ-tocopherol, 33% γ-tocotrienol and 24% δ-tocotrienol. Phenylhydrazine was freshly prepared in normal saline. Vitamin E-free palm oil was obtained from Golden Hope Bioganic, Selangor, Malaysia.

Male Wistar rats (200–250 g) were supplied by the Laboratory Animal Resource Units, Faculty of Medicine, Universiti Kebangsaan Malaysia. They were housed two rats per polyethylene cage sized 45×28×20 cm. The commercial rat chow (Gold Coin Ltd., Malaysia) and water were provided *ad libitum*. The rats were acclimatized for a week before the treatment was started.

### Experimental Design

Twenty-four male Wistar rats (180–220 g) were divided into two groups. The first group was given palm TRF at the dose of 30 mg/kg/day intraperitoneally [13], and the second group was administered vitamin E-free palm oil as a vehicle (control). The treatment was given for 14 days. Twenty-four hours after the last dose, each group was further divided into another two small groups; a group was administered phenylhydrazine (100 mg/kg, intraperitoneally) [16] and another group was administered normal saline. After twenty-four hours, blood and liver were collected for measurements of plasma total bilirubin, oxidative stress biomarkers and hepatic enzymatic activities. The study protocol and animal handling procedures in this study were approved by the Universiti Kebangsaan Malaysia Medical Research and Animal Ethics Committee (approval no. 295-March-2010-December-2010).

### Plasma Total Bilirubin Determination

Blood plasma was obtained from non-lyzed blood samples by centrifugation at 3000 rpm at 4°C for 10 minutes. Plasma total bilirubin was measured using a commercial kit supplied by Randox Laboratories Ltd. (UK). The analysis was performed using an automated Selectra E analyzer (Vitalab, US).

### Measurement of Red Blood Cell Lipid Peroxidation

The blood was collected using K_2_ EDTA-containing tubes. The blood was then centrifuged at 3000 g for 10 minutes at 4°C. The supernatant plasma was removed and packed red blood cells (RBC) were resuspended in phosphate buffered saline (0.1 M, pH 7.4). The cell suspension was then washed thrice and the supernatant aspirated.

Oxidative damage in RBC was freshly determined by measuring the thiobarbituric acid reactive substances (TBARS) as described by Grattagliano et al. [17]. Two hundred microliter of lyzed RBC (a part of RBC in nine parts of distilled water) was added to 2 ml of 0.67% thiobarbituric acid dissolved in 10% trichloroacetic acid and 0.25 M HCl. The suspension was heated at 100°C for 5 minutes and then cooled, followed by centrifugation at 3000 g for 15 minutes. The supernatant was spectrophotometrically read at 535 nm. The level of TBARS for each sample was expressed as nmoles of malondialdehyde (MDA) formed/ml RBC using an extinction coefficient of MDA as 1.56×10^5^ M^−1^cm^−1^.

### Measurement of Liver Lipid Peroxidation

Lipid peroxidation in the liver was colorimetrically estimated by the reaction of thiobarbituric acid with malondialdehyde, a stable lipid peroxidation product, according to the method of Ledwozyw et al. [18]. 1,1,3,3-Tetraethoxypropane was used as the standard and lipid peroxidation was expressed as thiobarbituric acid reactive substance (TBARS) in pmoles of MDA formed/mg protein. Briefly, 0.5 ml liver homogenate was added to 2.5 ml trichloroacetic acid (1.22 M) in 37% hydrochloride acid in a test tube. After incubation for 15 minutes at room temperature, the mixture was mixed with 1.5 ml 67% thiobarbituric acid in 0.05 M NaOH and then placed in 100°C water bath for 30 minutes. After cooling, 4 ml n-butanol was added to the mixture which was then vigorously vortexed and centrifuged at 3000 rpm for 10 minutes. The absorbance of butanol (supernatant) was measured using a UV spectrophotometer (Shidmadzu, UV-160A Japan) at 532 nm and expressed as pmol MDA/mg proteins.

### Measurement of Antioxidant Enzymes Activities

Superoxide dismutase (SOD) activity in the RBC hemolysate and liver homogenate were determined spectrophotometrically at 560 nm by the method of Beyer and Fridovich [19]. This method assay calculated the inhibition percentage of blue formazon formation (from the reaction of nitrobluetetrazolium and superoxide radical produced via the reduction of riboflavin) by the activity of SOD, and the results were expressed as U/mg protein. Catalase activity in the RBC hemolysate and liver homogenate were colorimetrically assayed by the decomposition of hydrogen peroxide at 240 nm using the method of Aebi [20]. One catalase unit was defined as 1 µmol of H_2_O_2_ consumed per minute. Glutathione peroxidase (GPx) activity in the RBC hemolysate was determined by the method of Lawrence and Burk [21]. The results of GPx activity were expressed as nmol NADPH oxidized/mg protein/min.

### Hepatic Aminolevulinic Acid Synthase Activity

Aminolevulinic acid synthase was assayed using a modified method from the one described by Marver et al. [22] in whole liver homogenate in 0.1 mM Tris-HCl buffered saline (pH 7.4) containing 0.5 mM EDTA. The reaction was started in the mixture which contained 0.1 M glycine, 0.01 M EDTA and 0.08 M Tris-HCl (pH 7.2) by an addition of the homogenate to a final volume of 2 ml. The reaction mixtures were incubated in flasks with shaking in air, at 37°C for an hour in dark before terminated by addition of 0.5 ml of trichloroacetic acid (25%). Later, the supernatant was obtained and 1 ml of it was added into 0.25 ml sodium acetate (2 M) and 0.5 ml acetyl acetone before incubated at 100°C for 15 min. After cooling, 1.3 ml Erhlich’s reagent in perchloric acid and glacial acetic acid containing 12.9 mM HgCl_2_ was added and read spectrophotometrically at 552 nm. The enzyme activity was calculated using an extinction coefficient of 5.8×10^4^ M^−1^cm^−1^.

### Hepatic Heme Oxygenase Activity

Heme oxygenase activity measurement was performed according to a modification to the method described by Taylor et al. [23]. Liver homogenate in 2 mM MgCl_2_-containing phosphate buffer (100 mM, pH 7.4) was centrifuged at 13,000 rpm. A volume of 100 µl sample supernatant (2–6 mg protein) was added to a reaction mixture that contained 2 mg protein of liver cytosol (a source of biliverdin reductase), 20 µM hemin, 0.8 mM NADPH, 2 mM glucose-6-phosphate and 0.002 unit/µl glucose-6-phosphate dehydrogenase and incubated at 37°C for an hour in dark. The final volume of the reaction mixture was 500 µl. The reaction was terminated by the addition of 500 µl chloroform. The chloroform lower layer was later read between 464 and 530 nm. The heme oxygenase activity was calculated based on extinction coefficient of 40 mM^−1^cm^−1^.

### Hepatic Biliverdin Reductase Activity

Liver microsomes were prepared prior to biliverdin reductase activity measurement following established methods [24], [25]. The enzyme activity was determined by the method of Niittynen et al. [25]. A reaction mixture with a total volume of 3.08 ml containing Tris (10 mM, pH 8), biliverdin (10 µM), and 2–6 mg/ml protein of sample cytosol was immediately mixed and read at 460 nm for a minute to record the blank rate. Then, 20 µl of β-NADPH (100 µM) was added and read again at 460 nm for a minute. The enzyme activity was calculated based on molar absorption coefficient of 52,500 M^−1^cm^−1^.

### Hepatic UDP-Glucuronyltransferase Activity

UDP-Glucuronyltransferase activity was determined in the liver microsomes following an established procedure [26]. A volume of 0.5 ml Tris-HCl buffer (0.15 M), 0.2 ml uridine diphosphoglucuronic acid (14 mM) and 0.2 ml p-nitrophenol (2 mM) were mixed and incubated in a shaking water bath at 37°C before an addition of 0.1 ml microsomal suspension (6–9 mg protein/ml) to start a reaction. After 10 minutes, the reaction was terminated by 2 ml trichloroacetic acid (18%). A volume of supernatant was mixed with the same volume of KOH (0.225 M). The sample was then read at 400 nm. The enzyme specific activity was calculated using p-nitrophenol molar absorption coefficient of 14.5×10^3^ M^−1^cm^−1^.

### Protein Content

The protein content in the samples was estimated using Lowry et al. [27] method. Bovine albumin serum was used as the standard and read at 700 nm.

### Statistical Analysis

Statistical analysis was performed by using SPSS 18.0 for Windows (SPSS, Chicago, USA). All values are expressed as mean ± standard error mean (SEM) of six animals. One-way analysis of variance (ANOVA), followed by Tukey multiple comparison test, was performed to compare the difference between the various treated groups. Values of p<0.05 were considered statistically significant.

## Results

### Effect on Plasma Total Bilirubin

**Figure 1:**
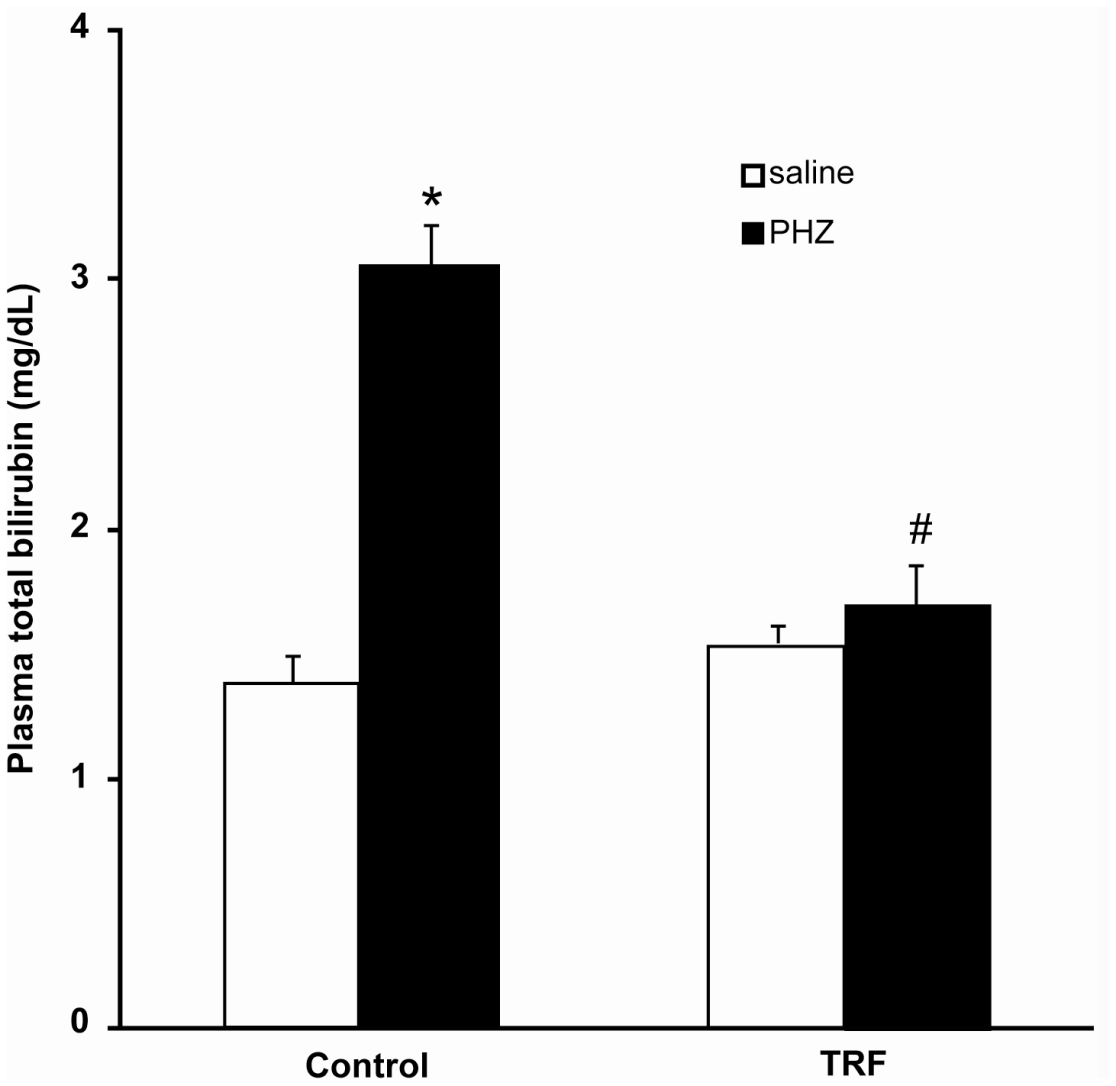
Plasma total bilirubin. Bars represent mean ± standard error (n = 6). *Different from the saline-treated group (P<0.005), #different from the control+PHZ (p<0.01). [TRF: tocotrienol-rich fraction; PHZ: phenylhydrazine].

A single dose of phenylhydrazine significantly increased plasma total bilirubin more than two-fold in rats (3.04±0.18 vs 1.37±0.11 mg/dl, p<0.005) (figure 1). Palm TRF pretreatment had significantly prevented the increase (1.69±0.16 vs 3.04±0.18 mg/dl, p<0.01). The level in the rats given palm TRF and phenylhydrazine was not different from the saline group (p>0.05).

### Effects on Oxidative Stress Biomarkers in Red Blood Cells

Administration of phenylhydrazine significantly increased TBARS content in the RBC. Palm TRF pretreatment was able to decrease the TBARS content in the rats given phenylhydrazine. However, the TBARS content in the palm TRF-pretreated group given saline was significantly lower than the same pretreated group given phenylhydrazine (table 1).

**Table 1:** Oxidative stress markers in red blood cells.

	TBARS (nmol MDA/ml)	SOD (U/mg protein)	Catalase (U/mg protein)	GPx (nmol/mg/min)
Control+saline	18.64±1.20	0.50±0.07	44.53±7.46	26.36±4.13
Control+PHZ	35.80±1.76*	0.21±0.03*	40.07±7.09	14.38±1.83
TRF+saline	17.72±1.80	0.42±0.07	38.96±8.40	25.79±9.38
TRF+PHZ	26.31±1.88*#	0.48±0.11#	39.92±7.46	18.47±3.40

Values are mean ± standard error (n = 6).

*Different from the saline-treated control (P<0.05),

# different from the control+PHZ (P<0.05). [TRF: tocotrienol-rich fraction; PHZ: phenylhydrazine].

Phenylhydrazine significantly reduced superoxide dismutase activity in the RBC. The activity of the enzyme in the palm TRF-pretreated group that was exposed to phenylhydrazine was significantly higher than the control group given phenylhydrazine. The enzyme activities in both palm TRF-treated groups (saline and phenylhydrazine) were similar. Both palm TRF and phenylhydrazine had no significant effects on the activities of catalase and glutathione peroxidase in the RBC. No other significant difference was noted (table 1).

### Effects on Oxidative Stress Biomarkers in Liver

The TBARS content in the liver was significantly higher in the phenylhydrazine-treated control group compared to the saline-treated rats. This increase was significantly decreased by the pretreatment of palm TRF. Both palm TRF-pretreated groups (saline and phenylhydrazine) had similar hepatic TBARS contents (table 2). The activities of hepatic superoxide dismutase and catalase in the rat livers were not affected by the palm TRF and phenylhydrazine.

**Table 2:** Oxidative stress markers in liver.

	TBARS (pmol MDA/mg protein)	SOD (U/mg protein)	Catalase (U/mg protein)
Control+saline	486.30±59.46	1.08±0.14	114.89±6.79
Control+PHZ	775.30±23.98*	1.09±0.07	114.89±6.28
TRF+saline	491.64±48.51	1.27±0.17	112.97±4.73
TRF+PHZ	616.49±17.50#	0.88±0.07	123.75±7.00

Values are mean ± standard error (n = 6).

*Different from the saline-treated control (P<0.001),

# different from the control+PHZ (P<0.001). [TRF: tocotrienol-rich fraction; PHZ: phenylhydrazine].

### Effect on Hepatic Bilirubin-Metabolizing Enzyme Activities

**Figure 2:**
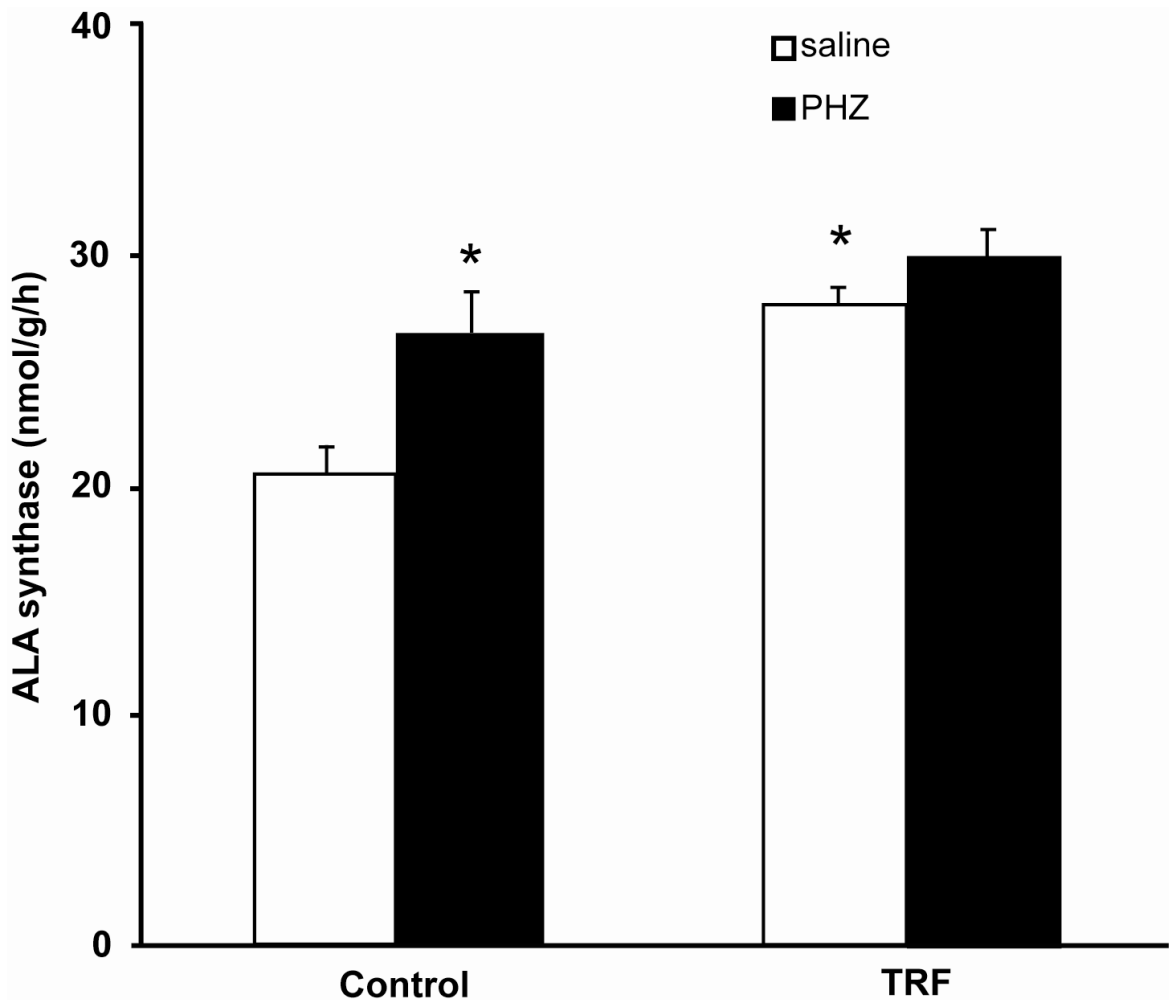
Hepatic aminolevulinic acid (ALA) synthase activity. Bars represent mean ± standard error (n = 6). *Different from the control+saline group (p<0.05). [TRF: tocotrienol-rich fraction; PHZ: phenylhydrazine].

Phenylhydrazine significantly elevated the activity of aminolevulinic acid synthase in the liver (figure 2). The enzyme activity was elevated in both groups pretreated with palm TRF (given saline and phenylhydrazine), which in the saline-treated group, the enzyme activity was significantly raised compared to the control group (27.98±0.67 vs 20.60±1.13 nmol/g/h, p<0.05).

**Figure 3:**
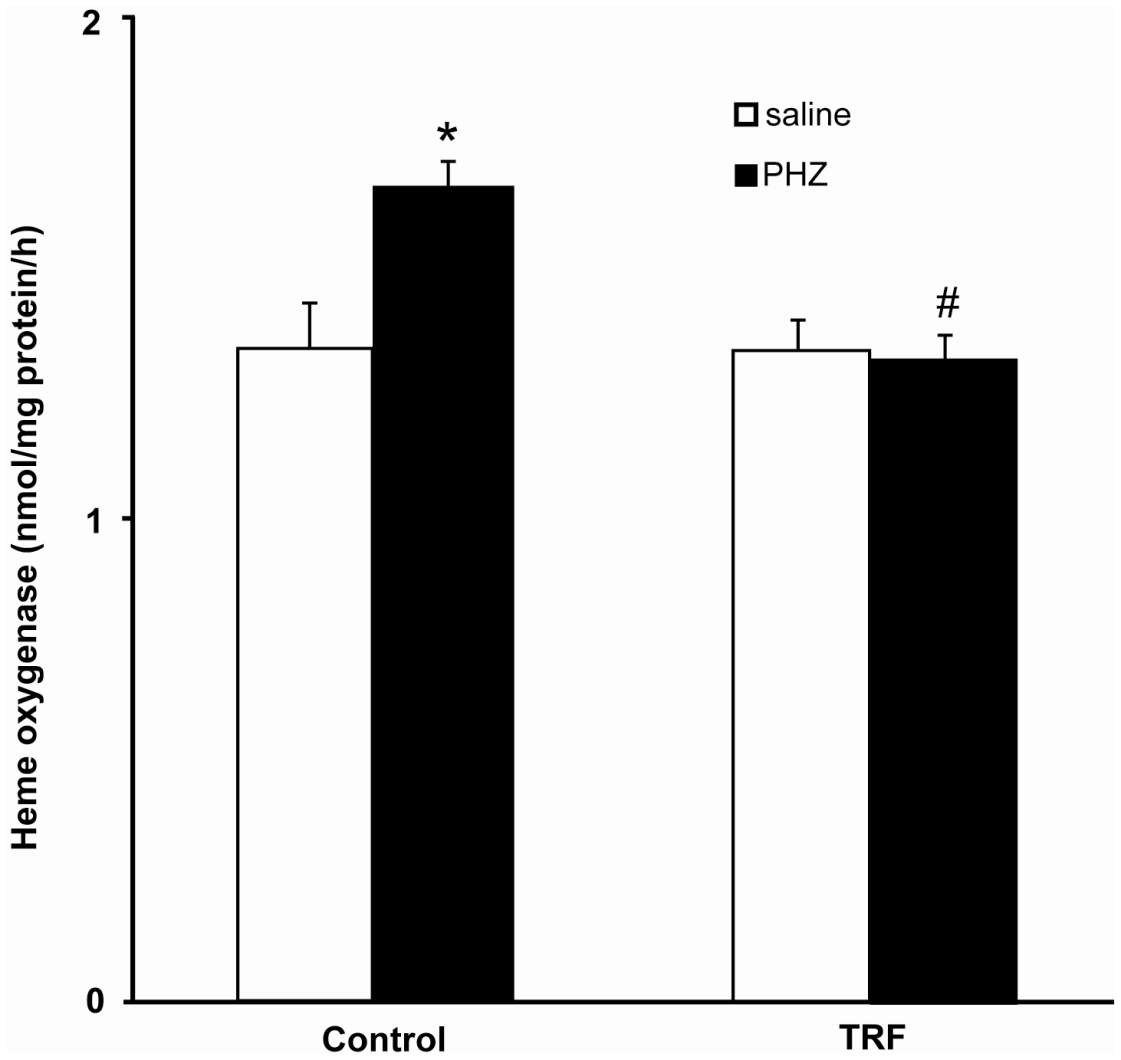
Hepatic heme oxygenase activity. Bars represent mean ± standard error (n = 6). *Different from the saline-treated group (p<0.05), #different from the control+PHZ (0.05). [TRF: tocotrienol-rich fraction; PHZ: phenylhydrazine].

**Figure 4:**
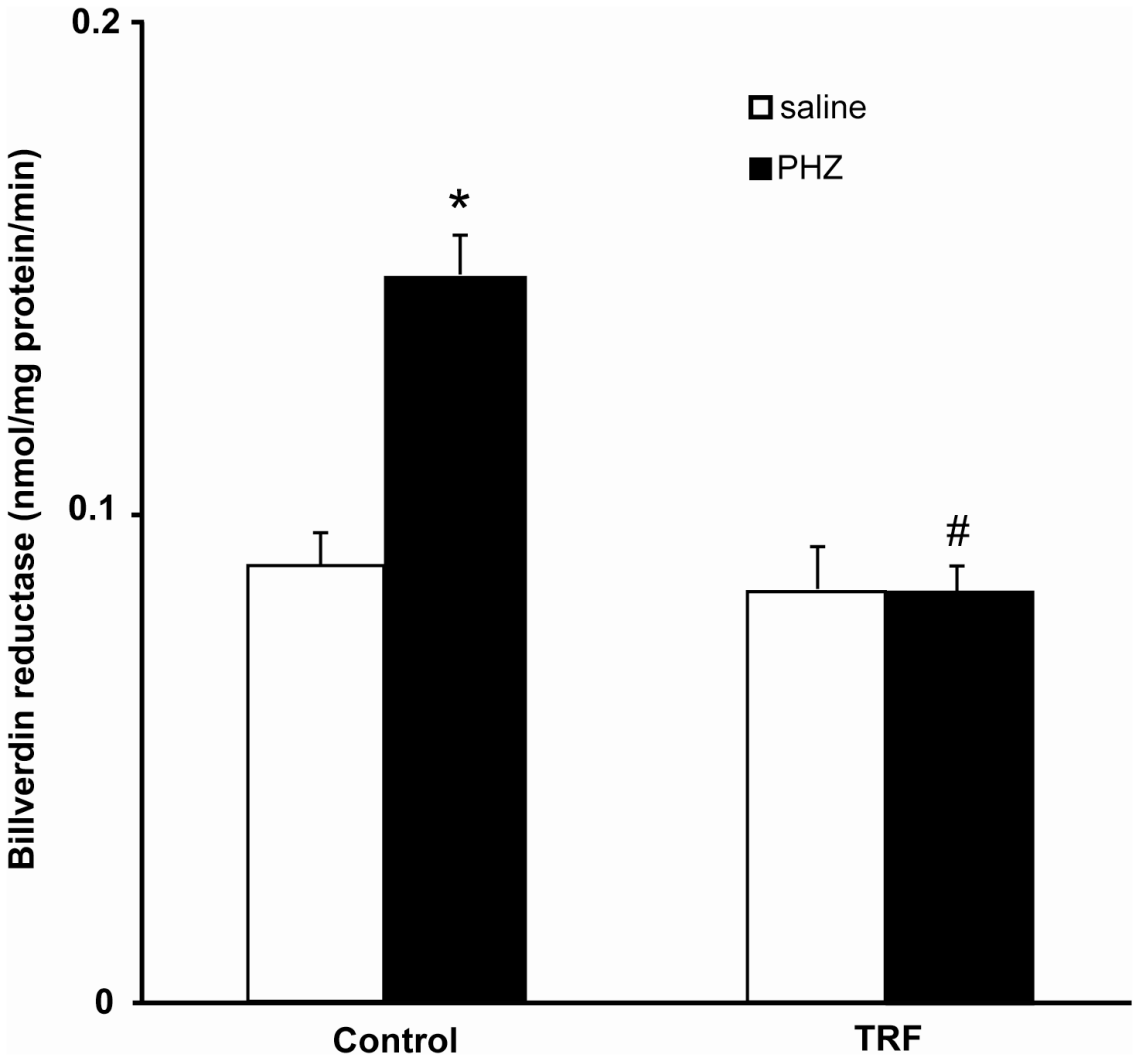
Hepatic biliverdin reductase activity. Bars represent mean ± standard error (n = 6). *Different from the control+saline group (p<0.05). [TRF: tocotrienol-rich fraction; PHZ: phenylhydrazine].

Figure 3 shows phenylhydrazine significantly increased hepatic heme oxygenase activity which was prevented by the palm TRF pretreatment. The enzyme activities in the palm TRF-pretreated groups (saline and phenylhydrazine) were not significantly different. Figure 4 shows the increase in phenylhydrazine-induced hepatic biliverdin reductase activity was prevented by the pretreatment of palm TRF.

**Figure 5:**
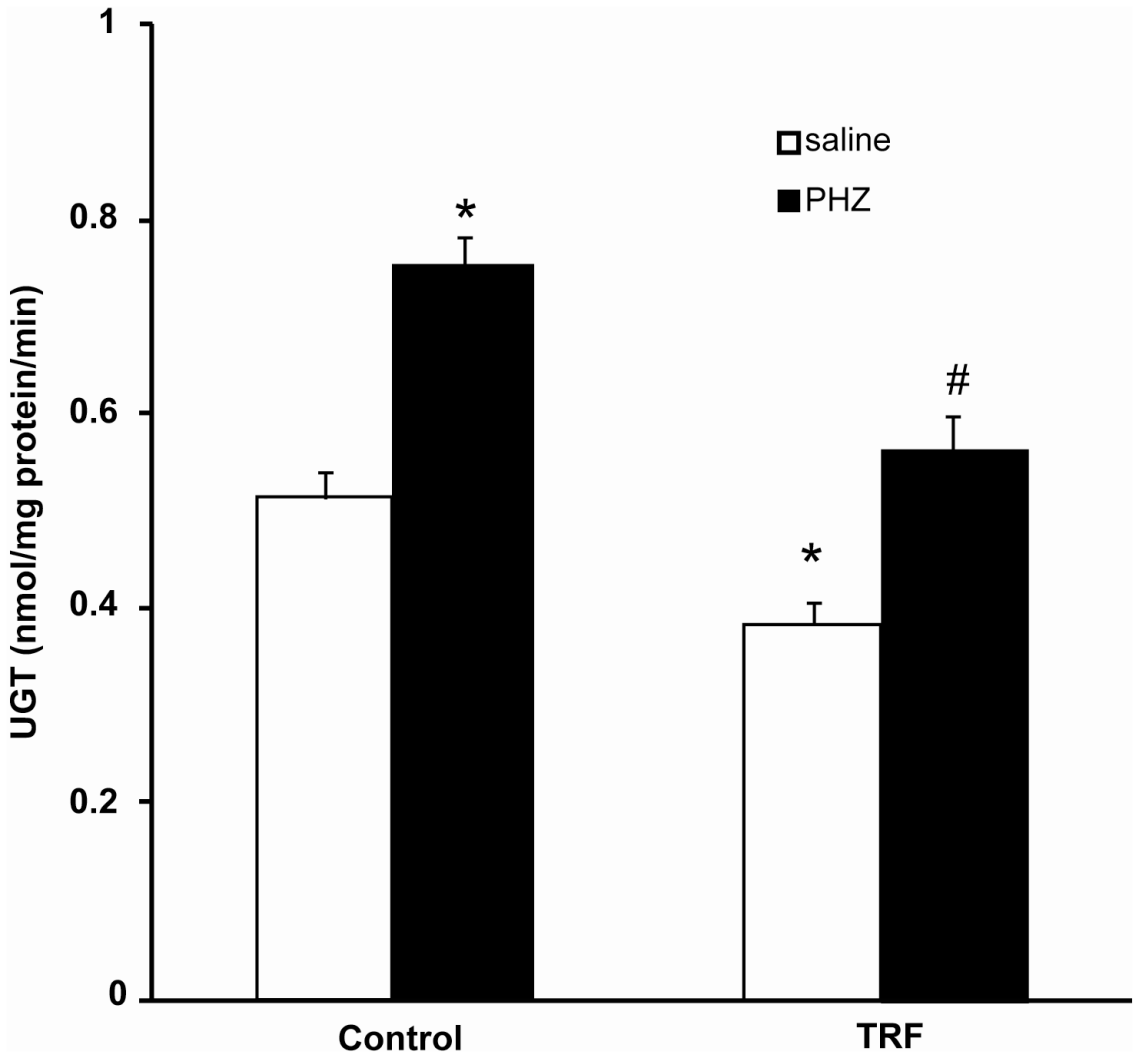
UDP-glucuronyltransferase activity in rat liver. Bars represent mean ± standard error (n = 6). *Different from the control+saline group; #different from the control+PHZ (p<0.05). [TRF: tocotrienol-rich fraction; PHZ: phenylhydrazine].

Hepatic UDP-glucuronyltransferase activity was significantly higher in the phenylhydrazine-treated control compared to the saline-treated group (figure 5). Palm TRF pretreatment had significantly prevented the increase. In the palm TRF group that was given saline, the activity was significantly lower than the saline-treated control (0.39±0.02 vs 0.52±0.02 nmol/mg protein/min, p<0.05).

## Discussion

An elevation of plasma total bilirubin following a single dose of phenylhydrazine was observed in this study. Phenylhydrazine produces reactive oxygen species and phenylhydrazine-derived radicals such as phenylhydrazyl radical, phenyldiazene and benzediazonium ions [4]. These reactive species would further promote oxidative destruction in the red blood cells which leads to hemolysis [28] and then increases the production of bilirubin [6]. Palm TRF on the other hand prevented the increase in plasma total bilirubin induced by phenylhydrazine. Our previous studies had demonstrated a similar protective effect of palm TRF on plasma total bilirubin elevation induced by δ-aminolevulinic acid [12], [13]. The protective effect could be due to the increased membrane stabilization effect by the vitamin in erythrocytes which would result in increased resistance to osmotic lysis [29].

In the present study, the increased content of TBARS in the red blood cells has confirmed the ability of phenylhydrazine to induce oxidative damage to the RBC.The increase in lipid peroxidation products is a result of free radicals activity that alters the lipid composition of the red blood cell membranes [30]. Increased oxidative stress was seen in rats with elevated heme oxygenase activity [9], which was also observed in our study. Increased lipid peroxidation induced by phenylhydrazine in the erythrocytes was accompanied by a decrease in superoxide dismutase activity, which was also reported by a recent study [5]. The reduction in the superoxide dismutase activity might be due to the oxidation of cysteine in the enzyme by superoxide anion during its transformation to hydrogen peroxide [31]. Phenylhydrazine also generates superoxide anion and hydrogen peroxide in addition to other free radicals [4]. Superoxide dismutaseconverts superoxide anion to oxygen and hydrogen peroxide, which is later detoxified by catalase and glutathione peroxidase [32]. However, the activities of the catalase and glutathione peroxidase in the red blood cells were unaffected by the phenylhydrazine administration in this study.Palm TRF had significantly reduced the increase in phenylhydrazine-induced lipid peroxidation content in the erythrocytes. It might be that the vitamin E had managed to scavenge some of the free radicals but still that was not enough to afford total protection against oxidative damage in the cells. However, it managed to totally reverse the detrimental effect of phenylhydrazine on the superoxide dismutase enzyme. Clinically, it can be used to assess the oxidative stress status in patients which increased activity, which may be suggestive of increased risk of hemolysis. It could be that the antioxidant property of the palm TRF had significantly reduced the oxidative load for the enzyme to handle, which managed to maintain its activity comparable to the control with saline. The antioxidant property of the palm TRF has been repeatedly reported in many studies [33], [34], [35]. The TRF had no effect on the catalase and glutathione peroxidase activities.

Phenylhydrazine raised lipid peroxidation content in the liver since the organ is the main site of iron storage [36]. The agent causes a striking increase in the free iron concentration deposited in the liver, due to the increased incidence of hemolysis [37]. Generated free radicals together with the released iron could further promote Fenton reaction which would induce lipid peroxidation in the liver. The oxidative damage was due to the reactive iron rather than the hepatic metabolism of the chemical [36]. However, phenylhydrazine had no effect on the antioxidant enzymes, namely superoxide dismutase and catalase. Palm TRF pretreatment reduced the lipid peroxidation content in the liver without affecting the antioxidant enzymes. This indicated that the TRF was able to reduce the oxidative damage in the liver induced by phenylhydrazine. It also reduced hepatic lipid peroxidation in δ-aminolevulinic acid-induced hyperbilirubinemic rat neonates [12], [13].

Phenylhydrazine induced the activity of aminolevulinic acid synthase, a rate-limiting enzyme for heme synthesis in the liver. The enzyme catalyzes the condensation of glycine and succinyl-CoA which produces aminolevulinic acid [38] and is regulated by cellular heme content [25]. Induction of the hepatic enyzme following an exposure to phenylhydrazine, was reported to be accompanied by a reduction in hepatic glutathione content, an endogenous antioxidant and an increase in heme content [8]. These findings suggest that increased heme level due to phenylhydrazine-induced hemolysis stimulates the hepatic aminolevulinic acid synthase and increases oxidative stress, which is in agreement with our study.

In the present study, it seemed that TRF had a direct stimulatory effect on the aminolevulinic acid synthase activity, regardless of phenylhydrazine administration due to the elevated enzyme activity in both groups pretreated with TRF. It is possible that TRF increased the synthesis of the enzyme or reduced its degradation. Further study is warranted to confirm this postulation. No study so far had reported the effect of tocotrienol on the enzyme. α-Tocopherol on this other hand, failed to prevent the enzyme inhibition by CdCl_2_ [39].

The stimulatory effect of phenylhydrazine was also seen on the heme oxygenase, which is in agreement with many other studies [9], [39]. The enzyme is a rate-limiting enzyme for heme degradation or bilirubin synthesis [7]. Exposure to phenylhydrazine would induce the enzyme activity to accommodate the increased production of heme. The reversal effect of palm TRF on heme oxygenase activity might be due to its protective effect at the earlier events that prevented heme overload. It could be that the vitamin reduced the oxidative damage to the erythrocytes and liver induced by phenylhydrazine, thus reduced the formation of heme. Studies had shown that α-tocopherol reduced the expression and activity of heme oxygenase [9], [40].Similarly, γ-tocotrienol reduced the expression of heme oxygenase in myocardial ischemia-reperfusion injury, but no significant effect was observed with α- and δ-tocotrienols [41].

A similar trend was also observed in the effect of phenylhydrazine and palm TRF on biliverdin reductase. Biliverdin reductase converts biliverdin to bilirubin. Therefore, it can be postulated that increased activity of the enzyme might reflect the increased formation of bilirubin, the product of interest in this study. Phenylhydrazine was demonstrated to increase the activity of the hepatic biliverdin reductase with a reduction in glutathione content [42], indicating an increase in oxidative stress. However, α-tocopherol had no significant effect on the enzyme activity [42]. Studies regarding the effect tocotrienol on biliverdin reductase activity or expression is still lacking. We postulated that the reduced activity of the enzyme in palm TRF-treated rats might be due to the reduced available biliverdin as a result of decreased heme oxygenase activity. It regulates heme oxidation in the cells by controlling the breakdown of the heme and heme oxygenase-1 expression [43]. Its activity can be induced by oxidative stress [44]. Therefore, most probably no significant increased oxidative stress in the liver of rats supplemented with palm TRF had prevented the increase in the enzyme activity.

Exposure to phenylhydrazine increased production of bilirubin as evidenced by an increase in plasma total bilirubin. The newly formed bilirubin would be conjugated to glucuronides by UDP-glucuronyltransferase and the increased load of bilirubin elevated the enzyme activity, as seen in the present study. So far, no other published report regarding the effect of phenylhydrazine on the UDP-glucuronyltransferase activity. Palm TRF inhibited the activity of UDP-glucuronyltransferase in rats administered phenylhydrazine. In the saline-treated group, palm TRF had further significantly reduced the activity when compared to the saline-treated control. A similar finding was also reported which showed inhibition of the enyzme by palm TRF (also known as palmvitee) in hyperbilirubinemic rat neonates [12]. This finding suggests that palm TRF inhibited the hepatic glucuronidation regardless of the exposure to phenylhydrazine. Thus, the inhibition of the increased plasma total bilirubin by the palm TRF was most likely not through its effect on hepatic glucuronidation due to the significant reduced activity of the enzyme seen in the non-hyperbilirubinemic rats. It could be that the palm TRF competed with bilirubin for the binding sites in the UDP-glucuronyltranssferase, caused conformational changes in the enzyme which blocked the binding of the substrate or increased the degradation of the enzyme, thus reduced the enzyme activity.

## Conclusion

The present study showed that pretreatment of palm TRF decreased phenylhydrazine-induced hyperbilirubinemia in rats, possibly via its antioxidant mechanism and by inhibiting hepatic heme oxygenase and biliverdin reductase activities.
